# Case–control study of diarrheal disease etiology in individuals over 5 years in southwest China

**DOI:** 10.1186/s13099-016-0141-1

**Published:** 2016-11-16

**Authors:** Shun-Xian Zhang, Chun-Li Yang, Wen-Peng Gu, Lin Ai, Emmanuel Serrano, Pin Yang, Xia Zhou, Shi-Zhu Li, Shan Lv, Zhi-Sheng Dang, Jun-Hu Chen, Wei Hu, Li-Guang Tian, Jia-Xu Chen, Xiao-Nong Zhou

**Affiliations:** 1 National Institute of Parasitic Diseases, Chinese Center for Disease Control and Prevention, Shanghai, 200025 People’s Republic of China; 2 Key Laboratory for Parasitology and Vector Biology, MOH of China, WHO Collaborating Center for Tropical Diseases, National Center for International Research on Tropical Diseases, Shanghai, 20025 People’s Republic of China; 3 Yunnan Provincial Center for Disease Control and Prevention, Kunming, 650022 People’s Republic of China; 4 Center for Environmental and Marine Studies (CESAM), Departamento de Biología, Universidade de Aveiro, Aveiro, Portugal; 5 Servei d´Ecopatologia de Fauna Salvatge (SEFaS), Departament de Medicina i Cirurgia Animals, Universitat Autònoma de Barcelona (UAB), Bellaterra, Spain; 6 Department of parasitology, College of Medicine, Soochow University, Suzhou, 215123 People’s Republic of China

**Keywords:** Acute diarrhea, Bacteria, Virus, Enteric protozoa, Co-infection

## Abstract

**Background:**

Acute diarrhea is one of the major public health problems worldwide. Most of studies on acute diarrhea have been made on infants aged below 5 years and few efforts have been made to identify the etiological agents of acute diarrhea in people over five, especially in China.

**Methods:**

271 diarrhea cases and 149 healthy controls over 5 years were recruited from four participating hospitals between June 2014 and July 2015. Each stool specimen was collected to detect a series of enteric pathogens, involving five viruses (*Rotavirus* group A, RVA; *Norovirus*, NoV; *Sapovirus*, SaV; *Astrovirus*, As; and *Adenovirus*, Ad), seven bacteria (diarrheagenic *Escherichia coli*, DEC; non-typhoidal *Salmonella*, NTS; *Shigella* spp.; Vibrio cholera; *Vibrio parahaemolyticus*; *Aeromonas* spp.; and *Plesiomonas* spp.) and three protozoa (*Cryptosporidium* spp., *Giardia lamblia, G. lamblia*, and *Blastocystis hominis, B. hominis*). Standard microbiological and molecular methods were applied to detect these pathogens. Data was analyzed using Chi square, Fisher-exact tests and logistic regressions.

**Results:**

The prevalence of at least one enteric pathogen was detected in 29.2% (79/271) acute diarrhea cases and in 12.1% (18/149) in healthy controls (*p* < 0.0001). Enteric viral infections (14.4%) were the most common in patients suffering from acute diarrhea, followed by bacteria (13.7%) and intestinal protozoa (4.8%). DEC (12.5%) was the most common causative agent in diarrhea cases, followed by NoV GII (10.0%), RVA (7.4%) and *B. hominis* (4.8%). The prevalence of co-infection was statistically higher (*p* = 0.0059) in the case group (7.7%) than in the healthy control (1.3%). RVA–NoV GII (3.0%) was the most common co-infection in symptomatic cases.

**Conclusions:**

DEC was the most predominant pathogen in diarrhea cases, but it was largely overlooked because the lack of laboratory capacities. Because of the high prevalence of co-infections, it is recommended the urgent development of alternative laboratory methods to assess polymicrobial infections. Such methodological improvements will result in a better prevention and treatment strategies to control diarrhea illness in China.

**Electronic supplementary material:**

The online version of this article (doi:10.1186/s13099-016-0141-1) contains supplementary material, which is available to authorized users.

## Background

Diarrheal illness is still a serious public health problem that particularly affects individuals in middle and low income countries [1]. Diarrhea is still a major reason of attendance at health services and one of the general causes for hospital admission [2]. In addition, 1,400,000 million deaths are caused by diarrhea across all age groups, of which 700,000 deaths are over 5 years [1, 2].

The main enteric pathogens include a wide range of bacteria (e.g. diarrheagenic *Escherichia coli*, DEC; non-typhoidal *Salmonella*, NTS; *Shigella* spp.; *Vibrio cholera*; *Vibrio parahaemolyticus*; *Aeromonas* spp.; *Plesiomonas* spp.; *Campylobacter* spp.), virus (e.g. *rotavirus* group A, RVA; *norovirus*, NoV; *Sapovirus*, SaV; *astrovirus*, As; *adenovirus*, Ad; *enterovirus*.) and enteric parasites (e.g. *Cryptosporidium* spp.; *Giardia lamblia, G. lamblia*; *Entamoeba histolytica* and *Blastocystis hominis, B. hominis*) [3–8].

Most researches of enteric pathogens on individuals with and without diarrhea have been largely based on a single or few pathogen species [9–11]. However, co-infection is a common prevalence in diarrhea cases in such communities with poor food hygiene, low sanitation and contaminated water (35.0, 20.1, 13.0%, respectively) [6, 12, 13]. Co-infection, however, are also common in healthy patients (8.0, 5.3, 0.8%, respectively) [6, 12, 13]. Co-infection is of particular human health importance because pathogen species can interact within the host. Interactions within the host can have either positive or negative effects on each of the co-infecting enteric pathogen species. Under positive enteric pathogen interactions, diarrheal disease transmission and progression are enhanced [6, 12, 14, 15].

Infectious diarrhea is still one of the important public health problems in China. The reported infectious diarrhea is up to 70,000,000, and the reported incidence of infectious diarrhea is 55.9/10,000,000 annually in China listed by China Information System for Diseases Control and Prevention. Diarrheal illness incidence is located in top three of 39 notifiable infectious diseases [11, 16]. However, in many medical institutions, the lack of clinical microbiology laboratories and detection capabilities hamper the detection of etiological agents of gastroenteritis. As result, etiology of gastroenteritis in China is achieved in less than 5.0% of patients [11]. In addition, most of the diarrhea studies have been limited to children under 5 years and either bacterial or viral species [11, 17, 18]. Hence, the aim of the study was twofold: one was to investigate the etiology of diarrhea cases in people over 5 years and to assess patterns of co-infection among virus, bacteria and protozoa in patients from southwest China. This study will contribute to the effective control of acute diarrhea in the country.

## Methods

### Subjects of this study

Acute gastroenteritis patients were defined as those who had diarrhea over three times within 24 h with abnormal stool specimens (e.g. mucus stool, watery stool, loose stool or bloody stool) in accordance with the WHO standard [19]. Non-diarrheal subjects were defined as those who had no history of diarrhea symptom before 14 days and were recruited at the same time as diarrheal subjects.

### Specimen and data collection

The stool specimens were collected from acute diarrhea cases and healthy controls over 5 years in outpatient from four sentinel hospitals as follows: The First people’s Hospital of Yunnan Province, Kunming Children’s Hospital, The Pushan Community Health Service Center in Kunming, The First People’s Hospital of Yunnan Province, and The First Affiliated Hospital of Kunming Medical University. A sterile sampling cup was applied to collect stool sample, with the criterion that each stool must be greater than 3 g or 3 mL, then each stool specimen was delivered to the laboratory of Yunnan Provincial Center for Disease Control and Prevention in Cary-Blair (C-B) culture medium (Oxoid Ltd, Basingstoke, UK) within 12 h. The clinical (e.g. fever, abdominal pain, nausea, vomiting, dehydration and tenesmus) and basic epidemiological data (e.g. sex, age, residence and season) was collected with structured questionnaire by doctors or nurses. The present study was conducted from July 2014 to June 2015.

### Laboratory test for enteric pathogens

Each stool sample was divided into three aliquots (Additional file 1). The first one was used for isolating, culturing and identifying bacterial (DEC, NTS, *Shigella* spp., *Vibrio cholera, Vibrio parahaemolyticus, Aeromonas* spp. and *Plesiomonas* spp.), the second one detect viral pathogens (RVA; NoV; *astrovirus* As, and *Adenovirus,* Ad), and last to assess intestinal protozoa infection (*Cryptosporidium* spp., *G. lamblia* and *B. hominis*).

#### Bacterial detection

MacConkey agar (MAC, Oxoid Ltd, Basingstoke, UK) was used for culturing DEC, which was divided into five subtypes by their virulence genetic as following: enteroaggregative *E. coli* (EAEC), enterotoxigenic *E. coli* (ETEC), enteropathogenic *E. coli* (EPEC), enteroinvasive *E. coli* (EIEC) and enterohaemorrhagic *E. coli* (EHEC). The DEC subtypes were examined with quantitative PCR based on the previous literatures (Table 1) [20, 21]. Each stool sample was inoculated into the selenite brilliant green sulfa enrichment broth (Oxoid Ltd, Basingstoke, UK) for enrichment and then inoculated it onto Salmonella–Shigella agar (Oxoid Ltd, Basingstoke, UK) to detect NTS. In addition, each stool specimen was inoculated directly onto Salmonella–Shigella agar (Oxoid Ltd, Basingstoke, UK) to find *Shigella* spp. Moreover, each sample was inoculated onto alkaline peptone water (Oxoid Ltd, Basingstoke, UK) for enrichment, and then inoculated onto thiosulfate-citrate-bile salts-sucrose agar (Oxoid Ltd, Basingstoke, UK) to detect *Vibrio cholera, Vibrio parahaemolyticus*, *Aeromonas* spp. and *Plesiomonas* spp. For suspicious NTS, *Shigella* spp., *Vibrio cholera, Vibrio parahaemolyticus, Aeromonas* spp., *and Plesiomonas* spp. colonies. The systematic biochemical identification of was performed using the VITEK^®^ 2 Compact instrument (bioMerieux, Marcyl’Etoile, France). Detailed detection procedures are found in references [11, 17].

**Table 1:** The primers and reactions condition applied to detect enteric pathogens in this study

Enteric pathogens	Target gene	Primer (5′–3′)	Amplicon sizes (bp)	Remarks	Source
EPEC	eae	CCACGGTTTATCAAACTGATAACG	105	Each stool specimen was inoculated to MAC media to culture DEC at 37 °C for 18 h, And then ten putative DEC colonies were selected to mix with 150 μL water to extract DNA at 100 °C for 10 min, and then the 20 μL volume of qPCR system is composed of 10 μL master mix (Takara Bio Inc, Shiga, Japan), 1 μL forward primer (10 μmol), 1 μL reverse primer (10 μmol), 1 μL DNA template and 7 μL H_2_O. The cycling conditions for each subtype DEC was 95 °C for 5 min, 40 cycles of 95 °C for 5 s, 60 °C for 30 s. The fluorescence recorded was at the annealing stage	[20, 21]
EHEC	stx1	ACTTCTCGACTGCAAAGACGTATG	132		
		ACAAATTATCCCCTGAGCCACTATC			
	stx2	CCACATCGGTGTCTGTTATTAACC	93		
		GGTCAAAACGCGCCTGATAG			
ETEC	elt	TTCCCACCGGATCACCAA	62		
		CAACCTTGTGGTGCATGATGA			
	estA	CCTTTCGCTCAGGATGCTAAAC	128		
		CAGTAATTGCTACTATTCATGCTTTCAG			
	estB	CTTTCCCCTCTTTTAGTCAGTCAACT	137		
		GCAGTAAAATGTGTTGTTCATATTTTCTG			
EAEC	aggR	CAGCGATACATTAAGACGCCTAAAG	116		
		CGTCAGCATCAGCTACAATTATTCC			
EIEC	ipaH	ACCATGCTCGCAGAGAAACT	175		
		TCAGTACAGCATGCCATGGT			
RVA	VP6	GACGGVGCRACTACATGGT	382	RVA, NoV GI, NoV GII, SaV and As were RNA viruses, complementary DNA (cDNA) was synthesized using a random primer (Takara Bio Inc, Shiga, Japan) at 55 °C for 1.5 h, followed by 100 °C for 10 min, and holding at 4 °C. The reaction condition of RVA was 94 °C for 5 min, followed by 40 cycles at 94 °C for 1 min, 42 °C for 1 min, 72 °C for 1 min, and with final extension at 72 °C for 10 min. Multiplex RT-PCR was used to detect the presence of NoV GI, NoV GII, and SaV, the thermal profile consisted of 94 °C for 5 min, 40 cycles of 94 °C for 70 s, 49 °C for 70 s, and 72 °C for 1 min, followed by 72 °C for 10 min. The thermal profile of As was 94 °C for 5 min, 40 cycles of 94 °C for 30 s, 55 °C for 30 s, and 72 °C for 1 min, followed by 72 °C for 10 min	[22]
		GTCCAATTCATNCCTGGTGG			
NoV GI NoV GII SaV	Polymerase	TGACGATTTCATCATCACCATA	331/319		[23]
		TGACGATTTCATCATCCCCGTA			
		GATTACTCCAGGTGGGACTCCAC			
		GATTACTCCAGGTGGGACTCAAC			
		GATTACTCCAGGTGGGATTCAAC			
		GATTACTCCAGGTGGGATTCCAC			
As	Capsid	CAACTCAGGAAACAGGGTGT	449		[24]
		TCAGATGCATTGTCATTGGT			
Ad	Hexon	TTCCCCATGGCICAYAACAC	482	The thermal profile was 94 °C for 5 min, 40 cycles of 94 °C for 30 s, 55 °C for 30 s, and 72 °C for 1 min, followed by 72 °C for 10 min	[25]
		CCCTGGTAKCCRATRTTGTA			
*Blastocistis hominis*	SSU-rRNA	CGAATGGCTCATTATATCAGTT	260	The thermal profile was 94 °C for 5 min, 40 cycles of 94 °C for 30 s, 53 °C for 30 s, and 72 °C for 1 min, followed by 72 °C for 10 min	[26]
		TCTTCGTTACCCGTTACTGC			
*Cryptosporidium* spp.	18S-rRNA	TTCTAGAGCTAATACATGCG		The primary cycle consisted of 94 °C for 5 min, 35 cycles of 94 °C for 50 s, 55 °C for 1 min and 72 °C for 90 s, followed by 72 °C for 10 min, the annealing step for a second reaction was 58 °C	[27]
		CCCATTTCCTTCGAAACAGGA			
		GGAAGGGTTGTATTTATTAGATAAAG	840		
		CTCATAAGGTGCTGAAGGAGTA			
*Giardia lamblia*	Tim	AAATIATGCCTGCTCGTCG		The thermal profile of first round was 94 °C for 1 min, 53 °C for 1 min, and 72 °C for 1 min, followed by 72 °C for 10 min. A second reaction was carried out similarly	[28]
		CAAACCTTITCCGCAAACC			
		CCCTTCATCGGIGGTAACTT	530		
		GTGGCCACCACICCCGTGCC			

DEC is composed of EAEC, EPEC, EIEC, ETEC and EHEC in this study, the judging standard of subtypes of DEC according to qPCR was: EPEC: eae+; EAEC: aggR+; EIEC: ipaH+; EHEC: eae+, and (stx1+; and/or stx2+); ETEC: hlt+, and/or estA, and/or estB+

#### Virus detection

Nucleic Acid was extracted from each stool specimen (15% wt/vol or vol/vol suspension) with QIAamp Viral RNA Kit (Qiagen, Hilden, Germany). The reverse transcription-polymerase chain reaction (RT-PCR) was applied to detected RVA [22], NoV (GI, GII) [23] and As [24]. For RT, the viral RNA was reverse transcribed with PrimeScript™ RT reagent Kit (Takara Bio Inc, Shiga, Japan). Ad was found using PCR [25] (Table 1).

#### Enteric protozoan detection

The genomic DNA of *Cryptosporidium* spp., G. *lamblia* and *B. hominis* was extracted from each stool sample with QIAamp DNA stool mini kit (Qiagen, Hilden, Germany) according to the manufacturers’ protocol. The conventional PCR was applied to detect *B. hominis* [26], the nested PCR was used to detect *Cryptosporidium* spp. [27] and G. *lamblia* [28] (Table 1).

### Data analysis

Data analysis was performed by IBM SPSS software (version 19.0 for Windows, Armonk, NY). Odds ratio (OR) and 95% CIs of categorical variables were calculated using two tailed Chi square or Fisher’s exact tests. Quantitative variable was described as mean, median, standard deviation or interquartile range (IQR), among which the median or mean of quantitative variable was compared by rank-sum test, analysis of variance or t test. Logistic regression was performed to find the relationship between diarrhea illness and various enteric pathogens. Single etiology was selected according to bivariate analysis with *p* < 0.20. Significant difference was taken as the level of *p* < 0.05 with two-tailed test.

## Results

### Basic information and clinical symptoms

From July 2014 to June 2015, 420 subjects were recruited for this study, which including 271 diarrhea cases and 149 healthy controls over 5 years. The male-to-female ratio was 0.964 in diarrhea cases and 0.961 in healthy controls (χ^2^ < 0.001, *p* = 0.987), respectively. The median age was 40.0 years in acute diarrhea cases and 41.4 years in non-diarrheal group(*t* = 0.817, *p* = 0.414). The diarrhea cases from urban areas accounted for 67.9%, and the non-diarrhea patients accounted for 66.4% (χ^2^ = 1.240, *p* = 0.538). The subjects in the 5–15 years age group was 64.5% in diarrhea cases and 63.1% in healthy controls (χ^2^ = 0.767, *p* = 0.681). The most frequent clinical symptom was nausea (*n* = 91, 33.6%) in diarrhea cases, and other common symptoms included abdominal pain (*n* = 73, 26.9%), vomiting (*n* = 58, 21.4%) and fever (*n* = 22, 8.1%). Mucus stool (*n* = 173, 63.8%) was the most common stool type in diarrhea cases, followed by watery stool (*n* = 70, 25.8%) and other types of stool (*n* = 28, 10.3%) (Table 2). The frequency of diarrhea was 5.8 times in acute diarrhea cases within 24 h (Additional file 2).

**Table 2:** Basic information and clinical characteristics of 271 acute diarrhea cases and 149 controls over 5 years

Characteristic	Diarrhea	Control
	n (%)	n (%)
n	271	149
Age		
5–15 years	21 (7.7)	9 (6.0)
15–50 years	175 (64.6)	94 (63.1)
≥50 years	75 (27.7)	46 (30.9)
Sex		
Male	133 (49.1)	73 (49.0)
Female	138 (50.9)	76 (51.0)
Residence		
Urban	184 (67.9)	99 (66.4)
Rural–urban fringe zone	68 (25.1)	35 (23.5)
Rural	19 (7.0)	15 (10.1)
Seasons		
Spring (Feb–Apr)	87 (32.1)	32 (21.5)
Summer (May–Jul)	65 (24.0)	42 (28.2)
Autumn (Aug–Oct)	59 (25.5)	45 (30.2)
Winter (Nov–Jan)	50 (18.5)	30 (37.5)
Symptom		
Fever (>37.3 °C)	23 (8.5)	–
Abdominal pain	73 (26.9)	–
Nausea	91 (33.6)	–
Vomiting	58 (21.4)	–
Dehydration	3 (1.1)	–
Tenesmus	5 (1.8)	–
Diarrhea		–
Watery stool	70 (25.8)	–
Mucus stool	173 (63.8)	–
Other stool	28 (10.3)	–

SD represent for standard deviation. Kunming city (25º 02′ 20″ N, 102º 43′05″ E, 1891 m.a.s.l.) has a humid subtropical climate of moderate seasonality characterized by a mild (mean temperature = 11.4 °C, min = 8, max = 15) and dry (mean precipitation = 33.4 mm, min = 12, max = 89) autumn (Aug–Oct) and winter (Nov–Jan). Spring (Feb–Apr) and summer (May–Jul) are also mild (mean temperature = 23 °C, min = 19, max = 29) but wet (mean precipitation = 159.6 mm, min = 92, max = 206) seasons. The “–” symbol indicates the information can not be collected

### The prevalence of enteropathogen in subjects with diarrhea or not

At least one enteropathogen was isolated from 79 (29.2%) of 271 acute diarrhea cases and 18 (12.1%) of 149 healthy controls (χ^2^ = 15.774, *p* < 0.0001). The overall prevalence of bacterial pathogen and viral pathogen in diarrhea cases were higher than in healthy controls (χ^2^ = 11.327, *p* = 0.001; χ^2^ = 10.795, *p* = 0.001 respectively. Table 3). At least one intestinal protozoa was found in 4.8% (*n* = 13) of cases and 6.0% (*n* = 9) of controls (χ^2^ = 0.299, *p* = 0.584) (Table 3). In univariate analysis, Details of the enteric pathogens isolates are presented in Table 3, and according to that EAEC, NoV and RVA were more prevalent (χ^2^ = 7.061, *p* = 0.008; χ^2^ = 9.160, *p* = 0.002; χ^2^ = 7.061, *p* = 0.008 respectively) in diarrhea patients (7.4, 10.0, 7.4%, respectively) than in healthy controls (1.3, 2.0, 1.3%, respectively, Table 3). No statistical difference was observed between acute diarrhea patients and healthy subjects for EPEC, ETEC, NTS, *Plesiomonas* spp., SaV, As, *B. hominis* and *Cryptosporidium* spp. In addition, other enteric parasites were not detected in subjects with and without diarrhea (Table 3). However, the multivariate analysis showed that only RVA was an enteric pathogen associated with diarrhea. But EAEC and NoV GII did not relate with diarrheal illness among individuals over 5 years (Table 3).

**Table 3:** Enteric pathogens in the stool samples with diarrhea cases (n = 271) and healthy controls (n = 149) in Kunming, China

Enteropathogen	Diarrhea cases n = 271 n (%)	Healthy controls n = 149 n (%)	Univariate analysis		Multivariate analyses	
			*p* value	OR (95% CI)	*p* value	OR (95% CI)
At least one enteropathogen	79 (29.2)	18 (12.1)	*p* < 0.0001	3.00 (1.71–5.23)	–	–
At least one enteric bacterial pathogens	37 (13.7)	5 (3.4)	*p* = 0.001	4.55 (1.75–11.85)	–	–
DEC	34 (12.5)	5 (3.4)	*p* = 0.002	4.13 (1.58–10.80)	–	–
EAEC	20 (7.4)	2 (1.3)	*p* = 0.008	5.86 (1.35–25.41)	*p* = 0.198	5.95 (1.33–26.63)
EPEC	15 (5.5)	3 (2.0)	*p* = 0.088	2.85 (0.81–10.01)	*p* = 0.107	2.86 (0.80–10.27)
ETEC	1 (0.4)	0 (0.0)	–	–	–	–
EIEC	0 (0.0)	0 (0.0)	–	–	–	–
EHEC	0 (0.0)	0 (0.0)	–	–	–	–
NTS	2 (0.7)	0 (0.0)	*p* = 0.541	–	–	–
*Plesiomona*s spp.	1 (0.4)	0 (0.0)	–	–	–	–
*Vibrio parahaemolyticus*	0 (0.0)	0 (0.0)	–	–	–	–
*Vibrio cholera*	0 (0.0)	0 (0.0)	–	–	–	–
*Aeromonas* spp.	0 (0.0)	0 (0.0)	–	–	–	–
*Shigella* spp.	0 (0.0)	0 (0.0)	–	–	–	–
At least one enteric virus pathogens	39 (14.4)	6 (4.0)	*p* = 0.001	4.00 (1.66–9.70)	–	–
NoV GII	27 (10.0)	3 (2.0)	*p* = 0.002	5.38 (1.60–18.06)	*p* = 0.0794	3.86 (0.85–17.48)
RVA	20 (7.4)	2 (1.3)	*p* = 0.008	5.86 (1.35–25.41)	*p* = 0.0166	4.50 (1.31–15.43)
NoV GI	1 (0.4)	0 (0.0)	–	–	–	–
SaV	1 (0.4)	0 (0.0)	–	–	–	–
As	0 (0.0)	1 (0.7)	*p* = 0.355	–	–	–
Ad	0 (0.0)	0 (0.0)	–	–		
At least one enteric parasite pathogens	13 (4.8)	9 (6.0)	*p* = 0.584	0.78 (0.33–1.88)		
*B. hominis*	13 (4.8)	9 (6.0)	*p* = 0.584	0.78 (0.33–1.88)	*p* = 0.412	0.68 (0.27–1.71)
*Cryptosporidium* spp.	1 (0.4)	0 (0.0)	–	–	–	–
*Giardia lamblia*	0 (0.0)	0 (0.0)	–	–	–	–

Including the co-infection of enteric pathogens in diarrhea cases and healthy subjects. The “–” symbol indicates the data can not be calculated

In diarrhea cases, DEC (12.5%, *n* = 34) was the most common pathogen, followed by NoV GII (10.0%, *n* = 27), RVA (7.0%, *n* = 20) and *B. hominis* (4.8%, *n* = 13).

### Temporal distribution of enteric pathogen in diarrhea cases

The prevalence of EAEC, EPEC, RVA and *B. hominis* showed strong seasonal variations (Table 4). The detection rate of EAEC in summer was higher than in winter (*p* = 0.0045), and the prevalence of EPEC in summer was higher than in winter (*p* = 0.0156). RVA was mainly prevalent in autumn and winter (*p* = 0.0015), and the prevalence peak of *B. hominis* was summer (*p* < 0.0001). NoV GII was not statistically different in four seasons (χ^2^ = 3.359, *p* = 0.341).

**Table 4:** The seasonal characteristics of mainly enteric pathogen isolated from diarrhea cases

Enteropathogen	Spring (Feb–Apr) n = 87 n (%)	Summer (May–Jul) n = 65 n (%)	Autumn (Aug–Oct) n = 69 n (%)	Winter (Nov–Jan) n = 50 n (%)	χ^2^	*p* value
EAEC	1 (1.2)	9 (13.8)	8 (11.6)	2 (4.0)	–	*p* = 0.0045
EPEC	7 (8.0)	7 (10.8)	1 (1.4)	0 (0.0)	–	*p* = 0.0156
RVA	4 (4.6)	0 (0.0)	8 (11.6)	8 (16.0)	–	*p* = 0.0015
NoV	5 (5.7)	7 (10.8)	10 (14.5)	5 (10.0)	3.359	*p* = 0.341
*B. hominis*	0 (0.0)	7 (10.8)	6 (8.7)	0 (0.0)	–	*p* < 0.0001

Including the co-infection of any enteric pathogens in diarrhea cases. The “–” symbol indicates that data be calculated with Fisher-exact tests

### Prevalence of enteric pathogens in diarrhea cases in different age group

Acute diarrhea cases were divided into different age groups, in which 21 (7.7%), 175 (64.6%) and 75 (27.7%) belong to age groups of 5–15, 15–50 and ≥50 years (Table 5). EPEC infection was the highest in the age group of 5–15 years (*p* = 0.031) (Table 5), but the prevalence of EAEC, RVA, NoV GII and *B. hominis* were not statistical difference among these three age groups (Table 5), respectively.

**Table 5:** Prevalence of enteric pathogens in diarrhea cases in different age groups

Enteropathogen	Total n = 271 n (%)	5–15 years n = 21 n (%)	15–50 years n = 175 n (%)	≥50 years n = 75 n (%)	χ^2^	*p* value
At least one enteropathogens	79 (29.2)	9 (42.9)	52 (29.7)	18 (24.0)	2.90	*p* = 0.234
At least one bacterium	37 (13.7)	5 (23.8)	27 (15.4)	5 (6.7)	5.41	*p* = 0.0668
At least one virus	39 (14.4)	4 (19.0)	23 (13.1)	12 (16.0)	0.748	*p* = 0.688
At least one parasite	13 (4.8)	1 (4.8)	10 (5.7)	2 (2.7)	–	*p* = 0.654
EAEC	20 (7.4)	1 (4.8)	16 (9.1)	3 (4.0)	2.56	*p* = 0.323
EPEC	15 (5.5)	4 (19.0)	9 (5.0)	2 (2.7)	–	*p* = 0.031
NoV	27 (10.0)	4 (19.0)	15 (8.6)	8 (10.7)	2.35	*p* = 0.309
RVA	20 (7.4)	2 (9.5)	13 (7.4)	5 (6.7)	0.198	*p* = 0.906
*B. hominis*	13 (4.8)	1 (4.8)	10 (5.7)	2 (2.7)	–	*p* = 0.654

Including the co-infection of any enteric pathogens in diarrhea cases. The “–” symbol indicates that data be calculated with Fisher-exact tests

### Co-infection of enteric pathogen in diarrhea cases and healthy cases

In this study, the prevalence of co-infection with more than one enteric pathogens was higher than in healthy controls (Table 6, *p* = 0.0059, OR = 6.17, 95% CI 1.43–26.71). In various co-infection cases, the co-infection with two enteric pathogens was more commonly detected in diarrhea patients than non-diarrhea subjects (Table 6, *p* = 0.0079, OR = 5.86, 95% CI 1.35–25.41). However, the prevalence of co-infection with more than three enteric pathogens in patents was as much as in healthy controls.

**Table 6:** The co-infection of enteric pathogens detected in diarrhea cases and healthy controls

Co-infections of enteric pathogens	Diarrhea cases n = 271 n (%)	Healthy controls n = 149 n (%)	*p* value	OR (95% CI)
Any two any enteric pathogens	20 (7.4)	2 (1.3)	*p* = 0.0079	5.86 (1.35–25.41)
Virus–virus	9 (3.3)	0 (0.0)	*p* = 0.0298	–
RVA–NoV GII	8 (3.0)	0 (0.0)	*p* = 0.0549	–
Bacteria–virus	5 (1.8)	0 (0.0)	*p* = 0.166	–
DEC–NoV GII	4 (1.5)	0 (0.0)	*p* = 0.302	–
DEC–RVA	3 (1.1)	0 (0.0)	*p* = 0.556	–
Bacteria–protozoan	3 (1.1)	2 (1.3)	*p* = 0.999	0.83 (0.14–5.00)
DEC–*B. hominis*	3 (1.1)	2 (1.3)	*p* = 0.999	0.83 (0.14–5.00)
Any three enteric pathogens	1 (0.4)	0 (0.0)	*p* = 0.999	–
DEC–RVA–*Cryptosporidium* spp.	1 (0.4)	0 (0.0)	*p* = 0.999	–
Total	21 (7.7)	2 (1.3)	*p* = 0.0059	6.17 (1.43–26.71)

Only co-infections with two pathogens found in at least 1% of diarrhea cases have been shown. The “–” symbol indicates the data can not be calculated

20 diarrhea cases of co-infections with two pathogens was identified, whereby two pathogens were identified, the prominent prevalence was virus–virus (45.0%, 9/20), followed by bacteria–virus (25.0%, 5/20) and bacteria–protozoan (15.0%, 3/20), and the other comprised co-infection was less common in diarrhea cases. The highest prevalence of co-infection in diarrhea cases was RVA–NoV GII (3.0%, *n* = 8), followed by DEC–NoV GII (1.5%, *n* = 4), DEC–RVA (1.1%, *n* = 3) and DEC–*B. hominis* (1.1%, *n* = 3). The prevalence of other co-infection between two pathogens was less than 1.0% in acute diarrhea cases (Table 6).

## Discussion

Since most studies had focused on diarrheal illness in children under 5 years [6, 11], little is known about the prevalence of acute diarrhea caused by enteric pathogens among person over 5 years. This study was the first of its kind conducted to determine the enteropathogens of acute diarrheal disease in Yunnan Province, China, and a series of pathogens involving bacteria, viruses and parasites were examined with a combination of conventional and molecular diagnostic techniques.

The detection rate of at least one enteric pathogen was significantly higher in diarrhea cases than in healthy controls, which showed a wide range of pathogens involving bacteria, and similar results have also been obtained from other countries [29, 30]. Although bacteria and parasites were the prominent enteropathogen in acute diarrheal cases aged more than 5 years in some developing countries [31], to our surprise, viral pathogens (RVA and NoV) were the most common pathogen in present study.

DEC were detected with a PCR method in stool sample from the patients and non-diarrheal controls, and the result showed that DEC wasn’t the causative agent of diarrhea in individuals over 5 years, and similar conclusions were shown in another study [32]. However, the authors of the other study argued that DEC was one of important enteric pathogen causing acute diarrhea [33]. The detection rate of DEC in present study was lower than that presented in other study [32], but it was higher than that presented in other region of China [11]. The prevalence of DEC varies greatly in different regions due to the detection method [11], behavior habits, geography and environmental hygiene among different areas [34]. Although the molecular biology techniques (e.g. PCR and Real-time PCR) are useful for detecting DEC, PCR was not used widely in medical facilities because of constraints in many developing countries, including the poor laboratory conditions, limited funds and low detection capacities of staff [16]. Hence, DEC was not a pathogen that was routinely detected in clinical laboratories especially in low and middle income countries [35, 36]. The DEC was detected in many studies with the traditional serum agglutination method which has low sensitivity and specificity. Therefore, the prevalence of DEC was underestimated and the pathogenic spectrum of acute diarrheal illness was not accurately described [4]. It was accurately described to detect DEC by PCR with high sensitivity and specificity due to the following reasons [31]: Firstly, the clinical symptom of diarrhea caused by different DEC subtypes and other enteropathogens cannot be distinguished easily. Secondly, DEC is widely prevalent in food and environment, and the modern tourism and trade had accelerated the spread of DEC. The modern detection method (e.g. PCR) can improve the sensitivity and specificity for detecting DEC in stool samples in order to accurately assess the burden of DEC in cases [4, 31]. In addition, the modern method has advantages in saving diagnosis time and reducing workload of finding DEC in diarrhea cases. Especially, it is more accurate to identify the various DEC subtypes, and it can be completed more quickly and more accurately.

EAEC is also the leading cause of diarrhea in children, adult and HIV-positive patients worldwide [37, 38]. In addition, EAEC was one of major causes of diarrhea outbreak in some developed countries (e.g. Europe, the UK and Japan) [31, 38]. EAEC was not the important bacterial pathogen associated diarrhea in individuals over 5 years in present study, and similar conclusion was obtained from other study [32]. However another study showed that EAEC was associated with diarrheal disease [33]. Further studies found that the concentration with 10^10^ CFU of serotype 042 EAEC strain can lead to diarrhea, but other serotype of EAEC strain cannot cause diarrhea in children and adults [37, 38]. It can be deducted that the genotype is likely to be an important factor in determining pathogenicity. The detection rate of EAEC in this study was as high as 7.4%, which was similar to the other study [33]. However the prevalence of EAEC was still lower than in many developing countries [37]. In the present study, EPEC was also not associated with diarrhea disease, similar to other study [31]. Further mechanism research might be conducted to explore the pathogenicity and infectivity at a genetic level.

Adults suffering diarrhea rarely visit a medical institution, unless they have acute serious or persistent diarrhea. The study suggests that although many enteric pathogens were detected from diarrhea patients over 5 years old, only RVA was significantly related with diarrheal illness in individuals over 5 years old. This study provides further evidence that RVA is a cause of acute adult diarrhea in China, but other study show that RVA was not an etiological agent with diarrhea [32]. The frequency of RVA infection (7.4%) was close to other study (9.6%) [39], but was higher than in the study (2.6%) conducted in adolescents or adults (10–89 years) in Italy [40].

NoV GII is one of major pathogens which can lead sporadic and outbreak acute diarrhea cases across all age groups worldwide [41]. The present study showed that NoV GII was the second most common enteropathogen in diarrhea cases. The high prevalence of NoV GII in individuals might be attributed to frequent social activities, and NoV GII is one of the most important food borne pathogen and exists widely in foods (such as shellfish, vegetables and water, et al.). These foods contaminated with NoV GII were primary reasons to lead sporadic and outbreak acute diarrhea [42–45]. The detection rate of NoV GII in our study was lower than that of in other study [46, 47]. The reason might be that seafood (e.g. shellfish) was not easily obtained and was not a conventional food in inland of China, including Kunming city.

*Blastocystis hominis* was found to be the most common protozoan in gastrointestinal tract of human and animals. It was widespread in natural world [48] and was highly prevalent in immunodeficiency patients [49]. *Blastocystis hominis* was not a pathogenic agent in present study, but other studies showed that *B. hominis* was a diarrhea-associated pathogen [50, 51]. *Blastocystis hominis* had high prevalence in healthy controls in present study implied that *B. hominis* was carried in health individual, which was a common phenomenon [50]. Whether *B. hominis* was one of pathogenic pathogen is need to explore the pathogenicity of different subtypes and mechanism. *Cryptosporidium* spp. and *G. lamblia* are leading cause of acute and chronic diarrhea in the tropics regions and some developing counties [52], but *Cryptosporidium* spp. had low prevalence and no one *G. lamblia* was detected in cases and healthy controls in present study, which indicating that these two kinds of intestinal protozoa were not serious disease burden and intimidate to individuals over 5 years old. This low prevalence of two protozoa might be due to epidemic characteristics of enteric parasites. Our research field was selected in urban with perfective municipal facilities of sewage treatment system, chlorine disinfection water, as well as, the population with high living level and health habits, so that the detection rate of enteric protozoa was very low, and the same results was showed in other studies in China [5, 17].

The co-infection was not neglected in diarrhea cases (7.7%) in this study, although other studies found that co-infection was high prevalent in sick individuals (13.0, 35.0, 25.0%, respectively) [12, 13, 53]. The co-infection leads to that individuals with greater levels of morbidity and mortality, making persons more vulnerable to species, for instance, the co-infection of RVA and other enteric pathogen can aggravate diarrheal symptom [14, 54]. In addition, the co-infection adds the difficulty to accurately determine etiological role of the enteric pathogen. Although co-infection by multiple groups of pathogens is the norm rather than the exception in nature, most research on the effect of pathogens on their hosts has been largely based on a single or few pathogen species [15]. Understanding the causes and consequences of co-infection among enteric pathogens remains one of the major challenges. Nevertheless, there is an increasing interest to move from the ‘diarrheal disease-one enteric pathogen’ perspective to a more holistic view of hosts as ecosystems of diarrhea illness [6], partially motivated by the health impact of co-occurring infections. In fact, in such complex ‘host–enteric pathogen ecosystems’ a variety of both direct and indirect interactions between enteric pathogens, their hosts and the circumstances must be taken into account [55].

## Limitations of this study

It was indentified several limitations in this study. Firstly, the study was conducted in an urban region that probably shows a poor representation of the potential enteric pathogen. Secondly, the diarrhea cases were selected from outpatients and hospitalized cases. But the patients who did not to seek medical advice were not recruited. Thirdly, helminthes and some intestinal bacteria were not detected in this study. Fourthly, the percentage of diarrheal patients who have taken antibiotics before the admission was not known, which may influence the detection rate of bacterial pathogens. In addition, enteric protozoa were not detected with microscopy, and the concentration of DNA in 1 μL can be different and therefore, the outcome of PCR might not be comparable [56]. Therefore, further research involved diarrhea case from urban, rural, outpatient and hospitalized might be done to evaluate the burden of diarrhea disease and assess the association between diarrhea and specific enteric pathogen. Match case–control study will be a good choice, and quantitate the DNA by nanodrop or something else and then loaded equal amount of DNA (e.g. 1 ng) for every PCR reaction will be have high reliability for entire project.

## Conclusions

Although it appears clear that RVA has impact on diarrhea illness, it was ignored in individuals over 5 years old. The prevalence of DEC was high in diarrhea cases, but it would be largely neglected due to lack of access to good quality diagnostic tests, which suggests that enhance laboratory capacities are urgently need in order to implement diarrhea surveillance programs. The co-infection was high prevalent in diarrhea cases, which will respond to better medical and public health interventions of diarrhea disease. In view of the diarrhea cases detected in urban region of Kunming city, Yunnan Province, which have effluent sewerage system, good sanitary condition and clean drinking water, it is concluded that food pollution might be the leading cause of acute gastroenteritis.

## Additional file

**Additional file 1:** The detection process of enteric pathogens applied in the study.

**Additional file 2:** The information of all subjects recruited in the study.

## Abbreviations

Ad: adenovirus
As: astrovirus
AWP: alkaline peptone water
*B. hominis*: *Blastocystis hominis*
CI: confidence interval
CFU: colony-forming unit-megakaryocyte
DEC: diarrheagenic *E. coli*
EAEC: enteroaggregative *E. coli*
EHEC: enterohemorrhagic *E. coli*
EIEC: enteroinvasive *E. coli*
EPEC: enteropathogenic *E. coli*
ETEC: enterotoxigenic *E. Coli*
*G. lamblia*: *Giardia lamblia*
MAC: macConkey agar
NoV: norovirus
NTS: non-typhoidal salmonella
OR: odd ratios
qPCR: quantitative PCR
RT-PCR: reverse transcription polymerase chain reaction
RVA: rotavirus A group
SaV: sapovirus
SD: standard deviation
SS: Salmonella–Shigella
TCBS: thiosulfate-citrate-bile salts-sucrose
WHO: World Health Organization
XLD: xylose, lysine and deoxycholate agar
