# Supplementary figures and images for: Case–control study of diarrheal disease etiology in individuals over 5 years in southwest China
Source: Gut Pathog. 2016 Nov 16;8:58. doi: 10.1186/s13099-016-0141-1 (PMC5112671; doi:10.1186/s13099-016-0141-1)

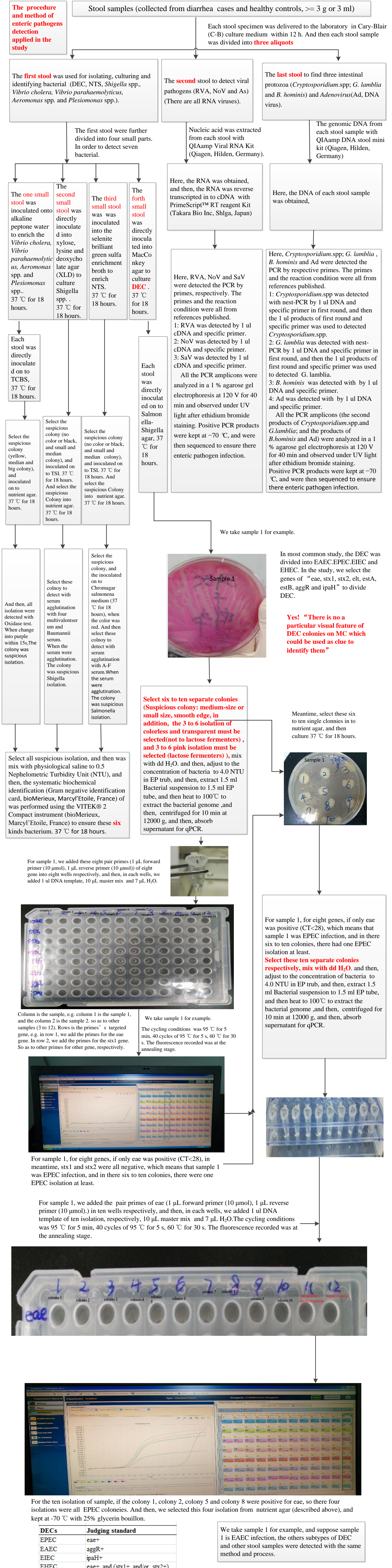

Supplement: Supplementary file 1 — Additional file 1: The detection process of enteric pathogens applied in the study. [file 13099_2016_141_MOESM1_ESM.pdf]
